# Health behaviours and psychosocial working conditions as predictors of disability pension due to different diagnoses: a population-based study

**DOI:** 10.1186/s12889-020-09567-8

**Published:** 2020-10-06

**Authors:** Annina Ropponen, Jurgita Narusyte, Karri Silventoinen, Pia Svedberg

**Affiliations:** 1 Division of Insurance Medicine, Department of Clinical Neuroscience, Karolinska Institutet, SE-171 77 Stockholm, Sweden; 2 Finnish Institute of Occupational Health, Helsinki, Finland; 3 Center of Epidemiology and Community Medicine, Stockholm County Council, Stockholm, Sweden; 4 Department of Social Research, Population Research Unit, University of Helsinki, Helsinki, Finland

**Keywords:** Cohort study, Physical activity, Sick leave, Health behavior, Musculoskeletal disorders, Disability pension

## Abstract

**Background:**

To investigate whether the clustering of different health behaviours (i.e. physical activity, tobacco use and alcohol consumption) influences the associations between psychosocial working conditions and disability pension due to different diagnoses.

**Methods:**

A population-based sample of 24,987 Swedish twins born before 1958 were followed from national registers for disability pension until 2013. Baseline survey data in 1998–2003 were used to assess health behaviours and psychosocial Job Exposure Matrix for job control, job demands and social support. Cox proportional hazards models were used to calculate hazard ratios (HR) with 95% confidence intervals (CI).

**Results:**

During follow-up, 1252 disability pensions due to musculoskeletal disorders (5%), 601 due to mental diagnoses (2%) and 1162 due to other diagnoses (5%) occurred. In the models controlling for covariates, each one-unit increase in job demands was associated with higher (HR 1.16, 95%CI 1.01–1.33) and in job control with lower (HR 0.87, 95%CI 0.80–0.94) risk of disability pension due to musculoskeletal disorders among those with unhealthy behaviours. Among those with healthy behaviours, one-unit increase of social support was associated with a higher risk of disability pension due to mental and due to other diagnoses (HRs 1.29–1.30, 95%CI 1.04–1.63).

**Conclusions:**

Job control and job demands were associated with the risk of disability pension due to musculoskeletal disorders only among those with unhealthy behaviours. Social support was a risk factor for disability pension due to mental or other diagnoses among those with healthy behaviours. Workplaces and occupational health care should acknowledge these simultaneous circumstances in order to prevent disability pension.

## Background

Along with efforts for sustainable working life [1], a growing interest is to avoid early exit from working life in terms of disability pension (DP) [2–4]. The number of DP is high: one in seven working age people rate themselves having chronic health problems or disability which affects daily life, and DP have also increased lately, despite improved health conditions [5, 6]. In the Nordic countries, DP constituted around 4% of GDP in 2015, whereas in OECD countries the level was around 2% [7]. A lot of research has been targeted to clarify the role of psychosocial working conditions in the risk of DP [8–11], but until recently, less attention has been paid on health behaviours and other factors potentially mediating or modifying these associations.

Existing literature of psychosocial working conditions as predictors for DP has yielded mixed results [10–14]. Often DP in general has been investigated, i.e. without assessing diagnosis for DP grant, or only one diagnosis group. The mixed results can be due to the limitation of data, e.g. occupational groups or industrial sectors [15, 16], but also the lack of power or no possibility to control for unmeasured confounding such as familial (genetics and shared environment) factors [10, 11].

Since DP is granted on medical grounds, an underlying disease plays an important role. Earlier studies have indicated that health behaviours, most importantly diet, physical activity, tobacco use, and alcohol consumption, increase the risk of preventable diseases [17, 18]. The assumed pathway is that even modest improvement in health behaviours could markedly affect the disease risk and consequently also the risk for DP. Thus, DP is known to be associated with various health behaviours [19–22] as has been also shown by earlier twin studies [23, 24]. Since many health behaviours are clustered and various other (i.e. occupational, behavioural) factors may also affect the associations with work incapacity in different ways, e.g. lack of physical activity may add the risk of work incapacity whereas non-smoking may decrease such a risk, the associations between health behaviors, working conditions and DP are complex and still poorly understood. A potential prevention strategy of many non-communicable diseases is to exert a simultaneous influence on several health behaviours [25, 26]. However, the question on the influence of working conditions and consequently on DP is still understudied.

Furthermore, the psychosocial working conditions might reflect genetic factors, e.g. through psychological factors with genetic background [27–29]. It is also known that genetics play a role in health behaviours including smoking [30], alcohol consumption [31] and sedentary physical activity [32], and on the risk of DP [33, 34]. Hence, twin studies provide a possibility to control familial factors and would be valuable when investigating the relations between psychosocial working conditions, health behaviours and DP. A co-twin control design in which discordant twins (i.e. one twin with DP and the twin sibling without DP) enables to ascertain whether the associations remain when familial factors have been controlled for. Utilizing the co-twin control design for the same-sex twins also provides match for age and sex in addition to familial factors.

The aim of this study was to investigate whether the clustering of different health behaviours (i.e. physical activity, smoking/oral tobacco and alcohol consumption) influences the associations between psychosocial working conditions and DP due to different diagnostic groups. We hypothesized that among those with healthy behaviours, the psychosocial working conditions would play a weaker role on the risk of DP than among those with unhealthy behaviours. In addition, we assumed the effect would be similar across various diagnosis groups. In specific, we investigated the effect of familial confounding on the associations.

## Methods

### Sample

The data were derived from the Swedish Twin project of Disability pension and Sickness absence (STODS) which is a prospective twin cohort study with the target population of all Swedish twins born in 1925–90. We restricted the final sample to those born 1935–1958 who at the time of the Screening Across the Lifespan Twin (SALT) study telephone interview were < 65 years old and not retired due to age or disability [35]. The analyses of discordant twin pairs included same-sexed twin pairs only (to control for sex). The Swedish Twin Registry conducted the SALT study between January 1998 and March 2003. There were 24,987 twin individuals in the final sample with complete data including 8439 complete twin pairs, 5344 same-sexed monozygotic and dizygotic pairs.

### Disability pension

All citizens in Sweden are included in a national social security scheme for DP. A medically confirmed disorder or injury permanently reducing work capacity by at least 25% is required for eligibility of DP. The thorough assessment of the level of work incapacity and consequent grant a DP is done by the Social Insurance Agency. DP benefit is taxable income and covers about 65% of lost income. For DP, date and diagnosis according to the International Classification of Diseases version 10 (ICD-10) were obtained from the National Social Insurance Agency Micro-Data for Analysis of the Social Insurance System (MiDAS) [36]. DP diagnoses were categorized as mental diagnoses (ICD-10 codes: F00-F99), DP due to musculoskeletal diagnoses (MSD, M00-M99), and DP due to other diagnoses (including all other diagnoses except mental diagnoses and MSD). The date of SALT (from 1 January 1998 to 31 March 2003) was the beginning of follow-up and the censoring date was the reaching 65 of age, old-age retirement (if earlier than age of 65), emigration (Statistics Sweden), death (the National Board of Health and Welfare), or on 31 December 2013, whichever occurred first.

### Psychosocial working conditions

Psychosocial working conditions were assessed via the Statistic Sweden’s Longitudinal integrated database for health insurance and labour market studies (LISA) data [36] in 1992. The psychosocial working conditions were based on the occupational codes as suggested by the Job-Demand-Control-Support model [37] through a validated psychosocial Job Exposure Matrix (JEM) [38] that has been reported earlier in detail [11, 39]. In short, the JEM is used to assign each occupation an age- and sex-specific mean score (range 1–10) for job demands, job control, and social support. A score of 10 indicates low demands, whereas score 10 is high for control and support. Instead a score of 1 indicates high job demands, but low control and support. We used these as continuous variables in the statistical analyses.

### Health behaviours

Health behaviours were based on physical activity, alcohol consumption and tobacco use from the SALT [40] and the past year frequency of leisure time physical activity was coded into three categories: high, moderate, and low [23]. Use of tobacco products (both smoking and oral tobacco) had four categories: never used (never or only tried), occasional user, regular user, and past user [41]. Alcohol consumption included self-reported consumed frequency and quantities of beer, wine, and spirits, and was calculated as reported in detail earlier [23], and the daily consumption of alcohol was categorized into four groups: abstainers, light, moderate, and heavy users [42]. Based on these health behaviour categories, a sum score for health behaviours was constructed using a binary score by the median split [23]. For the sum score, one point was received for each positive health behaviour (i.e. never used tobacco, moderate to high leisure-time physical activity and light alcohol consumption). For else, zero was marked. Then the points were summed to assign the health behaviour sum score from 0 (unhealthy behaviour) to 3 (very healthy behaviour), and for statistical analyses, dichotomized into unhealthy behaviours (scores 0–1) and healthy behaviours (scores 2–3). We had only a few missing cases (< 1%) in health behaviours except for alcohol consumption having missing data for 29%. Hence, in order to retain the power in the models, those with missing in alcohol were assigned with sex-specific mean value and included in the analysis to avoid losing that information.

### Covariates

In this study, we used sex, occupation, marital status, children living at home, and type of living area obtained from Statistics Sweden (LISA) [10] as covariates to be adjusted in the models. Occupation was included as covariate although it is a part of JEM since these were from different time points: JEM based on 1992 and occupation on 1990 data. Other covariates, self-rated health (SRH) and body mass index (BMI) were based on survey data. BMI was calculated from self-reports (weight in kilograms/ (height in meters) x (height in meters). SRH was queried with: “How would you rate your general health status?” and responses were collapsed into good, moderate, and poor.

### Statistical analyses

**Fig. 1:**
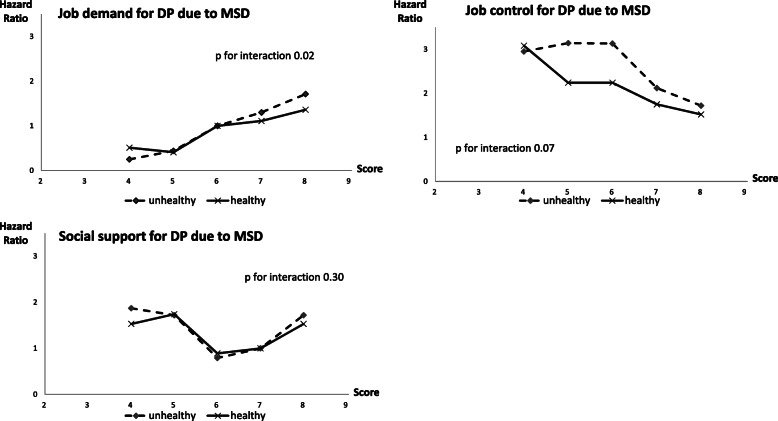
Hazard ratios (HR) for each value of psychosocial working conditions for the risk of DP due to MSD across healthy and unhealthy behaviours (due to large 95% CI no error bars are included in the figures to keep them readable). *P*-value is for interaction term

We conducted Cox proportional hazards regression models to calculate hazard ratios (HR) with 95% confidence intervals (CI) in which follow-up time was days and outcomes were DP due to MSD, mental diagnoses and all cause DP. The main exposures were psychosocial working conditions, job control, job demands, and social support assessed as continuous variables. No violation for the proportional hazards assumption was found when tested graphically or by Schoenfeld residuals (Figs. 1, 2, 3 and Table 1 in the Supplementary material). All the analyses based on the whole cohort were age- and sex-adjusted due to their important role in DP. Furthermore, all analyses were clustered on pair identity to adjust 95% CIs due to within pair dependency of twin data.

The effect of various covariates that were known to play a role for the risk of DP was taken into account: children living at home, marital status, SRH, occupation, and type of living area [23, 43]. We added them to a model at once (a full model). As we were specifically interested in the effect of health behaviours, we conducted stratified analyses for those with healthy vs. unhealthy behaviours.

Third, we tested the interactions between psychosocial working conditions and health behaviour categories in the age- and sex-adjusted model. We added the interaction term to the models adjusted for age and sex and tested the interaction term for statistical significance by the log-likelihood ratio test. We report the *p*-value for interaction term in the Fig. 1 to illustrate trends of psychosocial working conditions across healthy and unhealthy behaviours in association with DP due to MSD. For the illustration, we calculated HR for each value of psychosocial working conditions using the modal value as a reference (i.e. 6 for job demands and social support, 7 for job control).

We applied the co-twin control design by using conditional proportional hazards regression models. In the conditional model, the HRs were calculated using same-sex twin pairs discordant for DP, i.e. a twin in a pair had a DP while the co-twin had not been granted DP during the follow-up. The stratification by twin pair applies the follow-up time to DP in relation to the follow-up time of the co-twin to allow each twin pair to have their own baseline hazard. This design controls for potential confounding by familial factors (i.e. genetics and shared environment exist when twin pairs reared together share both their genetic make-up and home and family environment). If the association is due to familial factors, it should be found only between, but not within, twin pairs reared together, i.e., the association should exist in the analyses of the whole cohort but not in the conditional models. Analysing monozygotic and dizygotic twins separately provides understanding the role of genetic factors, which would be seen if the association were within dizygotic twin pairs who share about 50% of their segregating genes, but not within monozygotic twin pairs sharing 100% of genes. However, if the familial factors do not play a role, the association would exist within both monozygotic and dizygotic twin pairs. For statistics we used Stata version 14.2 MP (Stata Corporation, College Station, TX, USA).

## Results

Among the 24,987 individuals in the final sample, 1263 were granted a DP due to MSD (5%), 605 due to mental diagnoses (2%) and 1170 due to other diagnosis than MSD or mental diagnoses (5%) during the follow-up (mean follow-up time 8.3 years, SD 3.9). The descriptive characteristics are shown in Table 1.

**Table 1:** Descriptive statistics (frequency with percentage or mean with standard deviation, SD) for different DP diagnosis groups in the final sample of 24,987 Swedish twins

	DP due to MSD^1^ (*n* = 1252)				DP due to mental^2^ (*n* = 601)				DP due to other diagnoses^3^ (*n* = 1162)				No DP (*n* = 21,972)			
	Unhealthy behaviours (***n*** = 602)		Healthy behaviours (***n*** = 650)		Unhealthy behaviours (***n*** = 297)		Healthy behaviours (***n*** = 304)		Unhealthy behaviours (***n*** = 604)		Healthy behaviours (***n*** = 558)		Unhealthy behaviours (***n*** = 11,950)		Healthy behaviours (***n*** = 10,022)	
	N	%	N	%	N	%	N	%	N	%	N	%	N	%	N	%
Sex (women)	350	58	474	73	198	67	222	73	296	49	329	59	5161	43	5631	56
Marital status (living with someone)	425	71	448	69	179	60	167	55	410	68	382	68	8278	69	6956	69
Children living at home (yes)	402	67	447	69	206	69	202	66	380	63	388	70	8648	72	7149	71
Type of living area (semi-rural/rural)	226	38	272	42	77	26	97	32	166	27	226	41	3472	29	3491	35
Occupational groups																
Administration & management	61	10	60	9	51	17	45	15	91	15	86	15	2247	19	1752	17
Technology, natural and social science & art	44	7	45	7	62	21	79	26	102	17	88	16	2411	20	2015	20
Health care & social work	135	22	191	29	89	30	89	29	104	17	136	24	1924	16	2055	21
Commercial work	45	7	51	8	18	6	17	6	42	7	28	5	1046	9	668	7
Agriculture, forestry & fishing	21	3	30	5	3	1	4	1	14	2	13	2	315	3	429	4
Transport	43	7	29	4	13	4	13	4	38	6	31	6	602	5	453	5
Production & mining	174	29	144	22	42	14	33	11	151	25	121	22	2466	21	1764	18
Service & military work	79	13	100	15	19	6	24	8	62	10	55	10	930	8	886	9
Self-rated health																
Good	313	52	285	44	157	53	146	48	294	49	263	47	10,138	85	8243	82
Moderate	172	29	203	31	83	28	78	26	188	31	160	29	1513	13	1454	15
Poor	116	19	161	25	56	19	80	26	120	20	135	24	293	2	312	3
	mean	SD	mean	SD	mean	SD	mean	SD	mean	SD	mean	SD	mean	SD	mean	SD
Age at 1990 (years)	54.3	4.5	55.0	4.6	53.5	4.5	53.5	4.5	54.4	4.4	54.4	4.6	52.6	5.4	53.6	5.8
Job demands (range 1–10, high score is low)	6.3	0.7	6.3	0.8	5.8	0.8	5.9	0.8	6.0	0.7	6.1	0.7	5.9	0.7	6.0	0.7
Job control (range 1–10, high score is high)	6.1	1.2	6.1	1.2	6.7	1.2	6.6	1.1	6.6	1.3	6.5	1.3	6.9	1.2	6.4	1.3
Social support (range 1–10, high score is high)	6.4	0.7	6.6	0.7	6.5	0.5	6.6	0.5	6.3	0.6	6.5	0.6	6.3	0.6	6.4	0.6
Education in years	5.2	2.8	4.9	3.0	7.3	3.1	7.1	2.9	6.0	3.0	5.9	3.1	6.9	2.9	6.5	3.0
BMI	25.5	3.5	25.8	4.0	24.7	3.7	25.3	4.2	25.0	3.6	25.4	3.9	24.7	3.1	24.9	3.4

The comparison of healthy vs. unhealthy behaviours yielded mixed results. In the age- and sex-adjusted models, one-unit increase in job demands and job control predicted DP due to all studied diagnoses groups regardless of healthy or unhealthy behaviours. Instead, only among those with unhealthy behaviours, one-unit increase in job demands was associated with higher (HR 1.16, 95%CI 1.01, 1.33) and one-unit increase in job control with lower (HR 0.87, 95%CI 0.80, 0.94) risk of DP due to MSD when the covariates were controlled (Table 2). Among those with healthy behaviours, the one-unit increase of social support was associated with higher (HR 1.30, 95%CI 1.04, 1.63) risk of DP due to mental diagnoses and DP due to other diagnoses (HR 1.29, 95%CI 1.10, 1.53) when controlling for covariates.

**Table 2:** Cox proportional hazards ratios (HR) with 95% confidence intervals (CI) for associations between psychosocial working conditions and DP due to different diagnosis groups

	DP due to MSD (n = 1252)^b^							
	Age and sex adjusted model				Full model^a^			
	Unhealthy behaviours		Healthy behaviours		Unhealthy behaviours		Healthy behaviours	
	HR	95%CI	HR	95%CI	HR	95%CI	HR	95%CI
Job demands^e^	**1.84**	**1.67, 2.03**	**1.54**	**1.40, 1.69**	**1.16**	**1.01, 1.33**	0.95	0.83, 1.08
Job control^e^	**0.70**	**0.67, 0.74**	**0.77**	**0.73, 0.81**	**0.87**	**0.80, 0.94**	0.98	0.90, 1.06
Social support^e^	1.11	0.91, 1.36	1.17	0.97, 1.41	1.04	0.87, 1.26	1.12	0.93, 1.35
	**DP due to mental (*n*** **= 601)**^c^							
	**Age and sex adjusted model**				**Full model**^a^			
	**Unhealthy behaviours**		**Healthy behaviours**		**Unhealthy behaviours**		**Healthy behaviours**	
	**HR**	**95%CI**	**HR**	**95%CI**	**HR**	**95%CI**	**HR**	**95%CI**
Job demands^e^	**0.82**	**0.70, 0.97**	**0.82**	**0.71, 0.94**	0.83	0.68, 1.01	0.91	0.75, 1.10
Job control^e^	1.05	0.95, 1.17	**1.12**	**1.02, 1.24**	1.01	0.88, 1.15	1.06	0.93, 1.20
Social support^e^	1.13	0.88, 1.46	1.20	0.97, 1.49	1.10	0.85, 1.41	**1.30**	**1.04, 1.63**
	**DP due to other (*n*** **= 1162)**^d^							
	**Age and sex adjusted model**				**Full model**^a^			
	**Unhealthy behaviours**		**Healthy behaviours**		**Unhealthy behaviours**		**Healthy behaviours**	
	**HR**	**95%CI**	**HR**	**95%CI**	**HR**	**95%CI**	**HR**	**95%CI**
Job demands^e^	**1.25**	**1.13, 1.40**	**1.20**	**1.08, 1.33**	1.00	0.87, 1.16	1.05	0.91, 1.22
Job control^e^	**0.86**	**0.81, 0.91**	**0.91**	**0.85, 0.98**	0.96	0.88, 1.04	0.97	0.88, 1.07
Social support^e^	1.05	0.88, 1.26	**1.31**	**1.11, 1.55**	1.00	0.84, 1.18	**1.29**	**1.10, 1.53**

^a^Full model adjusted for age, sex, marital status, children living at home, type of living area, self-rated health, body mass index and occupational group

^b^DP due to MSD = disability pension due to musculoskeletal diagnoses

^c^DP due to mental = disability pension due to mental diagnoses

^d^DP due to other = disability pension due to any diagnoses excluding MSD and mental

^e^ Psychosocial working conditions are as continuous in the models i.e. HR is calculated for each one unit increase in score. For job demands a score of 10 indicates low demands, whereas score 10 is high for control and support. Instead a score of 1 indicates high job demands, but low control and support

The interactions between psychosocial working conditions and health behaviours were statistically significant (*p* < 0.05) only for job demands and DP due to MSD. The Fig. 1 illustrates the trends by the HR for each value of job control, job demands, and social support across healthy vs. unhealthy behaviours for the association with DP due to MSD.

Co-twin control analyses of discordant twin pairs showed attenuated associations between psychosocial working conditions and DP due to different diagnosis groups (Table 3). The results indicate that the effect of familial confounding cannot be ruled out, although for DP due to other diagnoses social support seem to be independent of familial confounding.

**Table 3:** Conditional Cox proportional hazards ratios (HR) with 95% confidence intervals (CI) for associations between psychosocial working conditions and DP due to different diagnosis groups

	DP due to MSD^c^					
	Discordant twin pairs (***n*** = 420)^a^		MZ pairs (***n*** = 178)		DZ pairs (***n*** = 242)	
	HR	95%CI	HR	95%CI	HR	95%CI
Job demands^b^	1.16	0.91, 1.48	1.46	0.88, 2.42	1.08	0.82, 1.43
Job control^b^	**0.83**	**0.72, 0.95**	0.84	0.66, 1.08	**0.82**	**0.69, 0.98**
Social support^b^	0.89	0.64, 1.23	1.08	0.59, 1.95	0.82	0.56, 1.21
	**DP due to mental**^d^					
	**Discordant twin pairs (*n*** **= 204)**^a^		**MZ pairs (*n*** **= 82)**		**DZ pairs (*n*** **= 122)**	
	**HR**	**95%CI**	**HR**	**95%CI**	**HR**	**95%CI**
Job demands^b^	0.85	0.59, 1.22	1.07	0.55, 2.06	0.76	0.48, 1.18
Job control^b^	1.03	0.84, 1.27	0.94	0.61, 1.45	1.6	0.83, 1.36
Social support^b^	1.09	0.63, 1.90	1.99	0.64, 6.13	0.85	0.44, 1.65
	**DP due to other**^e^					
	**Discordant twin pairs (*n*** **= 401)**^a^		**MZ pairs (*n*** **= 163)**		**DZ pairs (*n*** **= 238)**	
	**HR**	**95%CI**	**HR**	**95%CI**	**HR**	**95%CI**
Job demands^b^	1.00	0.71, 1.42	0.78	0.52, 1.15	1.14	0.82, 1.62
Job control^b^	1.02	0.89, 1.17	1.11	0.91, 1.35	0.95	0.80, 1.14
Social support^b^	1.00	0.70, 1.41	**0.47**	**0.24, 0.92**	1.40	0.91, 2.18

^a^ Only same-sexed complete pairs and adjusted for sum score of health behaviours, *MZ* monozygotic, *DZ* dizygotic

^b^ Psychosocial working conditions are as continuous in the models, i.e. HR is calculated for each one unit increase in score. For job demands a score of 10 indicates low demands, whereas score 10 is high for control and support. Instead a score of 1 indicates high job demands, but low control and support

^c^DP due to MSD = disability pension due to musculoskeletal diagnoses

^d^DP due to mental = disability pension due to mental diagnoses

^e^DP due to other = disability pension due to any diagnoses excluding MSD and mental

## Discussion

In this longitudinal twin cohort study with 24,987 participants, we investigated how the clustering of different health behaviours (i.e. physical activity, tobacco use and alcohol consumption) influences the associations between psychosocial working conditions and DP due to different diagnosis groups. Our results indicate that among those with healthy vs. unhealthy behaviours, the effect of psychosocial working conditions on the risk of DP varies, and the effect also varies between the diagnosis groups for DP. However, when accounting for several covariates (marital status, children living at home, type of living area, self-rated health, BMI and occupational group), most of the associations attenuated to statistical non-significance. Irrespective of the covariates, job demands and job control seem to play a more influential role among those with unhealthy behaviours for DP due to MSD, being along with our hypothesis. However, social support is important for DP due to mental or other diagnoses among those with healthy behaviours, not confirming our hypothesis. These results set new light on the earlier mixed findings of the associations between psychosocial working conditions and DP [10–14].

Our results that psychosocial working conditions predicted DP differently depending on DP diagnosis group confirms the importance to investigate the diagnosis groups instead of focusing on DP in general [12, 13] or only one diagnosis group [10, 11]. Furthermore, the assumption that health behaviours would be influential for the pathway to work incapacity in terms of DP [19–22] was confirmed in our study. In this study, the associations of job control, job demands and social support with DP varied if one had healthy or unhealthy behaviours based on tobacco and alcohol consumption and physical activity. Hence, we assume that this finding would be useful for preventive strategies at occupational health care or at workplaces in order to diminish the negative effects of psychosocial working conditions and to promote healthy lifestyle [23, 24, 43]. However, the attenuating effect of covariates is new since both the earlier study of health behaviours [23] and psychosocial working conditions [10, 11] have indicated independency from various covariates. It is possible that these factors are of interest for the mutual associations of health behaviours and psychosocial working conditions. Our results suggest that for DP due to MSD, attention should be paid on the excess of job demands to avoid DP and on providing more job control to enhance the risk decreasing effect if unhealthy behaviours co-exist. However, these should be accompanied with actions to promote healthy behaviours, e.g. support to quit smoking or initiation into physical activities. Instead for DP due to mental or other diagnosis, those with healthy behaviours might perceive social support as negative in terms of increasing the risk of DP. This finding requires further clarification as it may reflect some psychological constructs at workplaces (such as management and teams) [44] or related to individual characteristics (such as personality). Alternatively, this may reflect health awareness or being precautionary, in which social support might play a role both in health behaviours and in applying DP grant. In addition, genetic factors which are known to contribute on individual characteristics [45], behaviours [23] and social support [46], seem also to play a role in the association between social support and DP. Another aspect meriting further studies is the fact that we were able to detect statistically significant interaction between psychosocial working conditions and health behaviours only for DP due to MSD.

The unique aspect of this study was the access to twin cohort data that enabled us to investigate the unmeasured effects of familial confounding, i.e. genetics and shared, mainly childhood, environment, on the associations. Based on the earlier twin studies, familial confounding was known to exist in psychological factors [28], smoking [30], alcohol consumption [31], sedentary physical activity [32], and DP [33, 34]. However, due to limited number of discordant twin pairs (from 204 to 420), our results need to be interpreted with caution. Familial confounding cannot be ruled out, although social support seem to be independent from effects of familial confounding while analysing DP due to other diagnoses. This gives further support for the implication that different diagnosis groups for DP should be studied separately. Despite the cautious interpretation, our discordant twin pair results of limited familial confounding point to the direction that psychosocial working conditions and health behaviors need a greater emphasis at workplaces and occupational health care.

This large, population-based sample of twins with comprehensive survey and national register data have several strengths. First, the register data is complete without loss to follow-up and self-reporting bias including dates and diagnosis for DP. Second, our data enabled us to utilize the well-documented psychosocial JEM [38]. Third, we used sum score for health behaviours as they are known to co-occur, and to also have additive effects for DP [23]. Fourth, although our study may have lacked power in discordant twin pair analyses, the possibility to control for familial confounding adds to the other epidemiological studies relying on non-twin populations. Some limitations also merit attention. Despite the large sample size, the stratification on health behaviours and discordant twin pairs resulted in reduced sample size and lack of power suggesting that even larger sample sizes are needed to confirm these findings. We also had a relatively large amount of missing data for alcohol consumption and therefore imputed data with sex-specific mean for health behaviour sum score. That may have diluted our findings but as we used the median score for each behaviour the effect should be modest. Since underreporting of alcohol consumption is a common problem in self-reported data, this limitation might exist in other studies as well. Another limitation might be the fact that our data was based on a Swedish sample of middle-age twins, hence, findings might be less generalizable to other populations beyond Nordic countries and to younger cohorts. Last, the fact that our psychosocial working conditions were based on data from 1992 would imply that this study should be repeated with data of more recent measures. In specific, we need to mention that the health behaviours were measured in 1998–2003, which is six to 11 years after assessing psychosocial working conditions. Measuring both psychosocial working conditions and health behaviours at the same time would have been optimal, but we assume that our results would be more likely diluted than overestimated. However, keeping these limitations in mind, these results address the importance of mutual influence of psychosocial working conditions and health behaviours on the risk of DP due to different diagnosis groups relevant for preventive actions and strategies at occupational health care and at workplaces. As clinical implications this would suggest multidisciplinary approaches both at individual and at workplace level to support workability.

## Conclusions

Clustering of health behaviours played a differential role in the associations between psychosocial working conditions and DP depending on the diagnosis groups. The risk of disability pension due to musculoskeletal disorders was associated with job control and job demands only among those with unhealthy behaviours. Social support was a risk factor for disability pension due to mental or other diagnoses among those with healthy behaviours. Various confounding factors including familial ones play a role in these associations. Workplaces and occupational health care should acknowledge these simultaneous factors in order to affect psychosocial working conditions and health behaviours for prevention of permanent work incapacity.

## Supplementary information

**Additional file 1: Figure S1.** Disability pension due to musculoskeletal diagnoses, healthy vs. unhealthy behaviours. **Figure S2.** Disability pension due to mental diagnoses, healthy vs. unhealthy behaviours. **Figure S3.** Disability pension due to other diagnoses, healthy vs. unhealthy behaviours. **Table S1.** The proportional hazard assumption test using Schoenfeld residuals.

## Data Availability

The data cannot be made publicly available. According to the General Data Protection Regulation, The Swedish law SFS 2018:218, The Swedish Data Protection Act, the Swedish Ethical Review Act, and the Public Access to Information and Secrecy Act, these type of sensitive data can only be made available after legal review, for researchers who meet the criteria for access to this type of sensitive and confidential data. Readers may contact the last author regarding these details.

## Abbreviations

BMI: Body mass index
CI: Confidence intervals
DP: Disability pension
HR: Hazard ratio
ICD-10: International Classification of Diseases version 10
JEM: Job Exposure Matrix
LISA: Statistic Sweden’s Longitudinal integrated database for health insurance and labour market studies
MiDAS: National Social Insurance Agency Micro-Data for Analysis of the Social Insurance System
MSD: Musculoskeletal disorders
SALT: Screening Across the Lifespan Twin study
SD: Standard deviation
SRH: Self-rated health
STODS: Swedish Twin project of Disability pension and Sickness absence
STR: Swedish Twin Registry
