# Supplementary material for: Health behaviours and psychosocial working conditions as predictors of disability pension due to different diagnoses: a population-based study
Source: BMC Public Health. 2020 Oct 6;20:1507. doi: 10.1186/s12889-020-09567-8 (PMC7541297; doi:10.1186/s12889-020-09567-8)
Supplement: Supplementary file 1 — Additional file 1: Figure S1. Disability pension due to musculoskeletal diagnoses, healthy vs. unhealthy behaviours. Figure S2. Disability pension due to mental diagnoses, healthy vs. unhealthy behaviours. Figure S3. Disability pension due to other diagnoses, healthy vs. unhealthy behaviours. Table S1. The proportional hazard assumption test using Schoenfeld residuals. [file 12889_2020_9567_MOESM1_ESM.docx]

**Supplementary material**

The proportional hazards assumption tested graphically by observing the ‘log-log’ curves

**
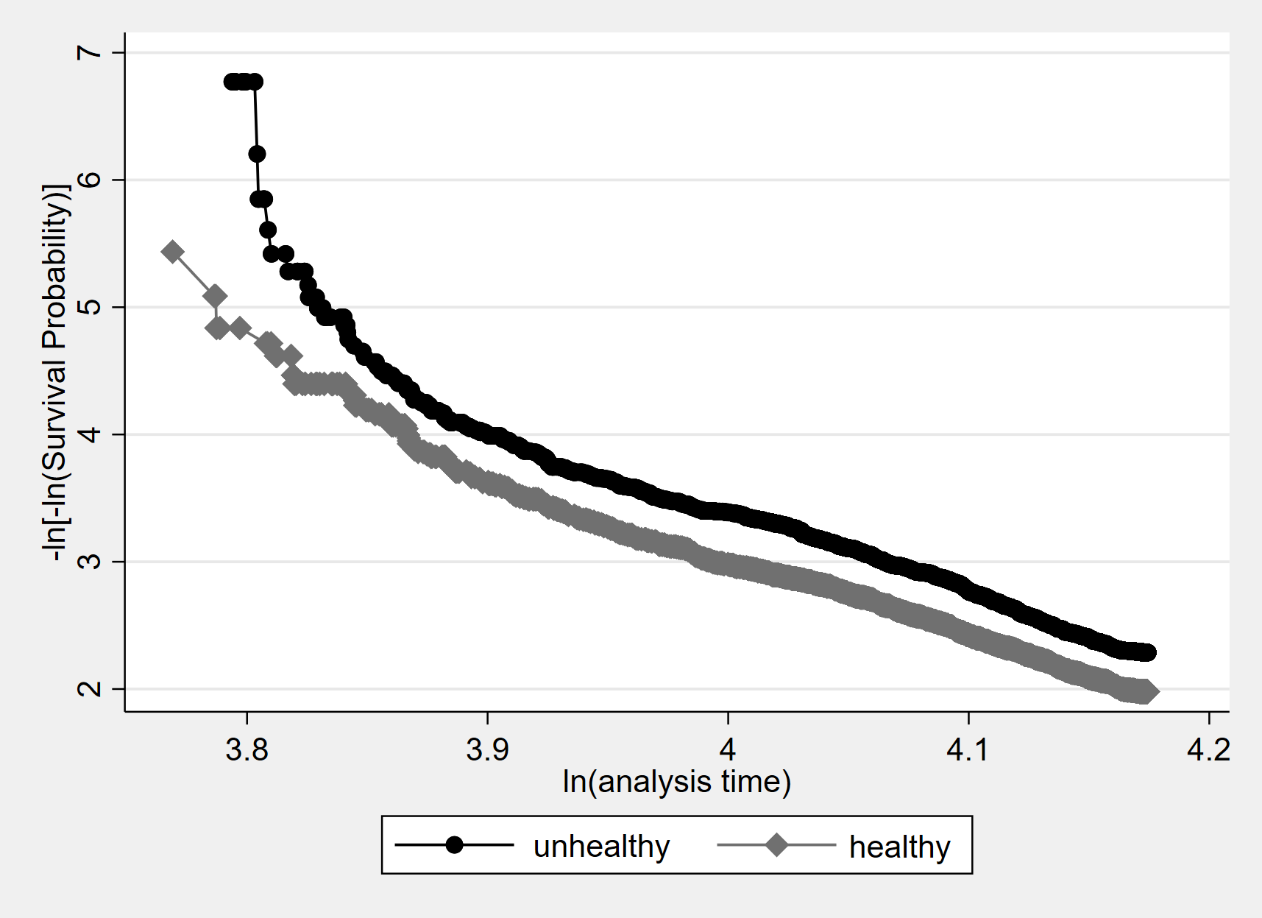
**

**Figure S.1** Disability pension due to musculoskeletal diagnoses, healthy vs. unhealthy behaviours

**
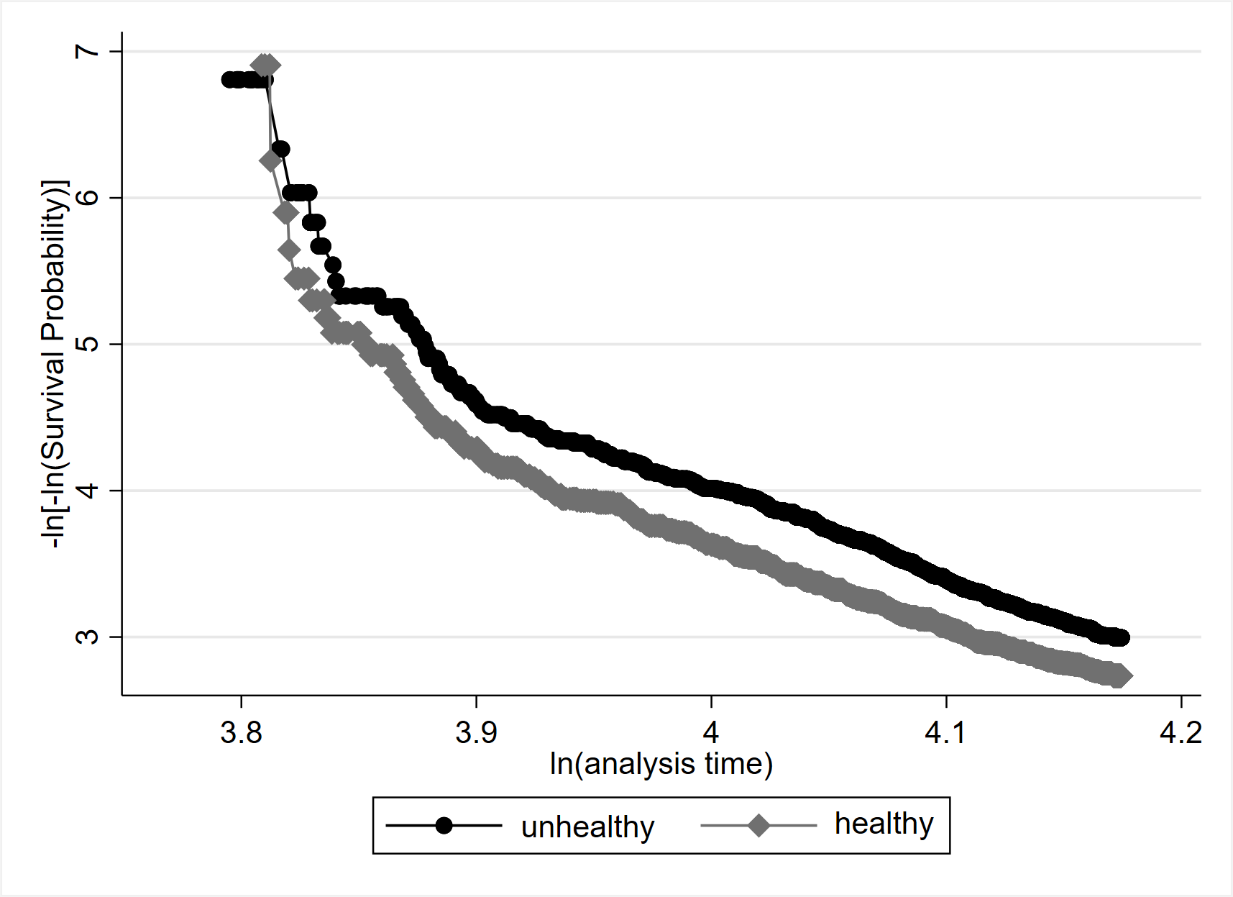
**

**Figure S.2** Disability pension due to mental diagnoses, healthy vs. unhealthy behaviours

**
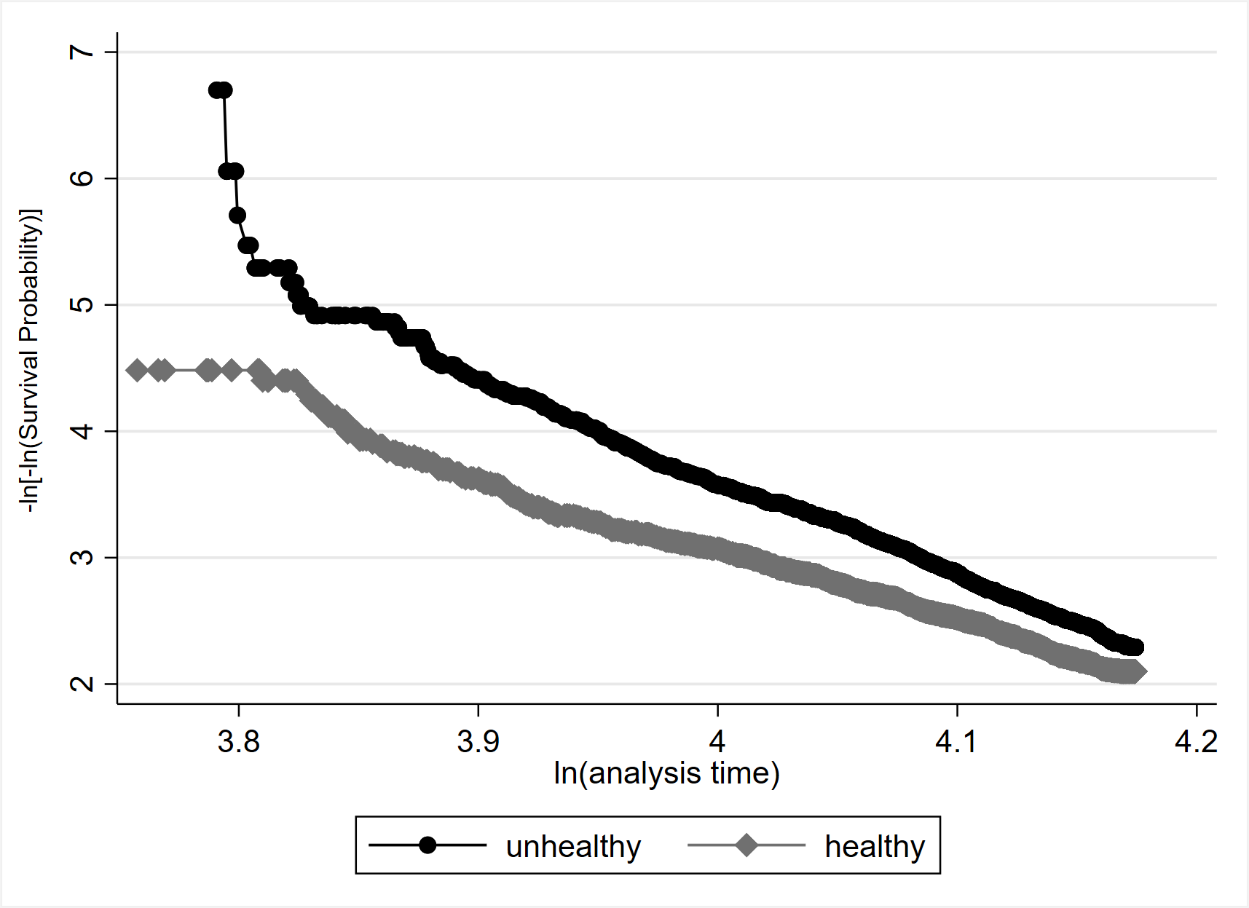
**

**Figure S.3** Disability pension due to other diagnoses, healthy vs. unhealthy behaviours

**Table S.1** The proportional hazard assumption test using Schoenfeld residuals

| Psychosocial working conditions | **DP due to MSD** | **DP due to mental** | **DP due to other** |
| --- | --- | --- | --- |
|  | **p-value** | **p-value** | **p-value** |
| Job demands | 0.66 | 0.89 | 0.17 |
| Job control | 0.19 | 0.80 | 0.09 |
| Social support | 0.16 | 0.59 | 0.09 |
